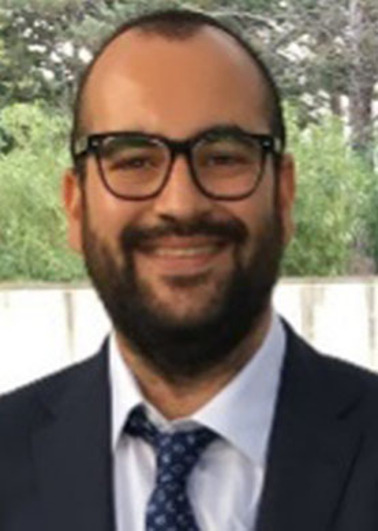# Amine‐Rich Carbon Dots as Novel Nano‐Aminocatalytic Platforms in Organic Synthesis

**DOI:** 10.1002/ejoc.202200879

**Published:** 2022-09-13

**Authors:** Vasco Corti, Beatrice Bartolomei, Martina Mamone, Giuseppe Gentile, Maurizio Prato, Giacomo Filippini

**Affiliations:** ^1^ Department of Chemical and Pharmaceutical Sciences INSTM UdR Trieste University of Trieste Via Licio Giorgieri 1 34127 Trieste Italy; ^2^ Centre for Cooperative Research in Biomaterials (CIC BiomaGUNE) Basque Research and Technology Alliance (BRTA) Paseo de Miramón 194 20014 Donostia San Sebastián Spain; ^3^ Basque Fdn Sci Ikerbasque 48013 Bilbao Spain

**Keywords:** Amines, Green chemistry, Nanostructures, Organocatalysis, Synthetic methods

## Abstract

The development of novel and effective metal‐free catalytic systems, which can drive value‐added organic transformations in environmentally benign solvents (for instance, water), is highly desirable. Moreover, these new catalysts need to be harmless, easy‐to‐prepare, and potentially recyclable. In this context, amine‐rich carbon dots (CDs) have recently emerged as promising nano‐catalytic platforms. These nitrogen‐doped nanoparticles, which show dimensions smaller than 10 nm, generally consist of carbon cores that are surrounded by shells containing numerous amino groups. In recent years, organic chemists have used these surface amines to guide the design of several synthetic methodologies under mild operative conditions. This Concept highlights the recent advances in the synthesis of amine‐rich carbon dots and their applications in organic catalysis, including forward‐looking opportunities within this research field.

## Graphical abstract

From *N*‐containing molecular precursors to amine‐rich CDs: a journey into sustainable and green opportunities for aminocatalytic methodologies. The analysis of the most explicative examples provides a panoramic view on the progress toward the preparation and application of amine‐bearing CDs as catalysts for transformations of synthetical importance.

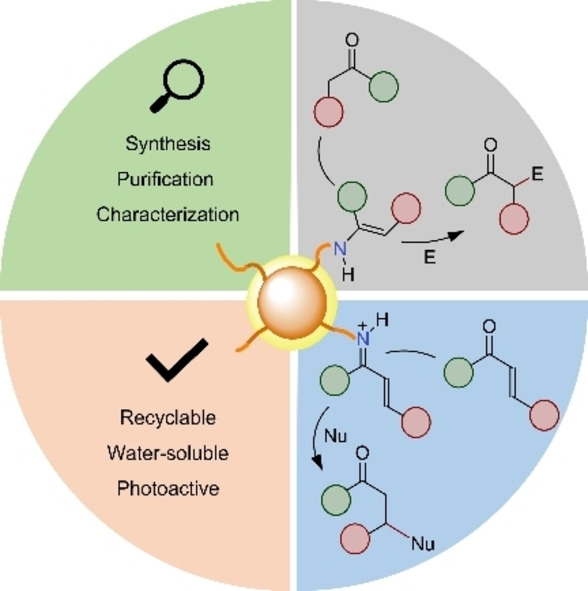

## 1 Introduction

**Figure 1:**
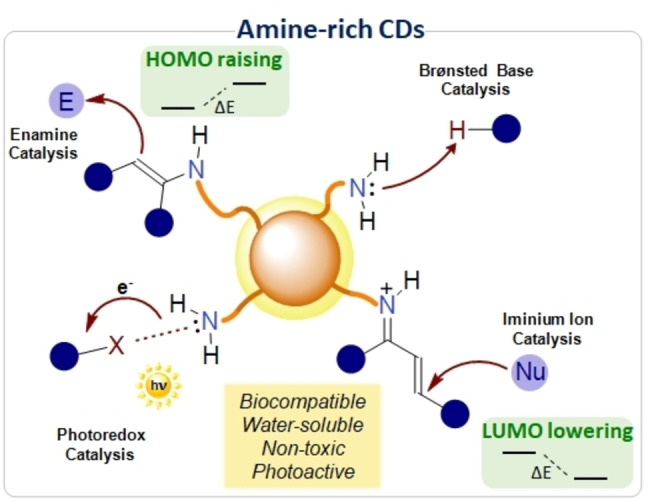
The application of amine‐rich CDs as nano‐organocatalysts in the field of enamine, iminium ion, Brønsted base and photoredox catalysis. E: electrophile; Nu: nucleophile; X: halogen atom.

Carbon Dots (CDs) are a relatively new class of carbon‐based nanomaterials with dimensions below 10 nm and quasi‐spherical shape.^[1]^ Over the years, these nanoparticles have gained popularity thanks to their interesting photophysical and optoelectronic properties.^[2]^ Besides, CDs do possess other appealing features, including low toxicity, biocompatibility and good to excellent solubility in water and common polar organic solvents.^[3]^ As for most of carbon nanomaterials, the appropriate selection of precursors and synthetic conditions are of pivotal importance to tailor their properties into specific applications.[^4^, ^5^] In particular, when molecules are used as precursors, the chemical structure of the resulting CDs may reflect the molecular features of the starting materials.[^6^, ^7^] As an example, by employing amino acids and amine‐bearing molecules it is possible to obtain nanoparticles that are rich in superficial amines and other nitrogen‐containing functionalities.^[8]^ Importantly, these surface amino groups are able to mimic the chemical behavior of classical molecular aminocatalysts, thus paving the way for the application of amine‐rich CDs as nano‐catalytic platforms in organic synthesis (Figure 1).^[9]^

Aminocatalysis, and more in general organocatalysis (i.e the use of low‐molecular‐weight molecules as catalysts for chemical transformations), is a well‐established research area, also recognized by the recent awarding of the Nobel Prize in chemistry.[^10^, ^11^, ^12^] Within the field of aminocatalysis, it is possible to identify two main activation modes: enamine and iminium ion catalysis.^[13]^ However, it is worth noting that aminocatalysts may be also used to activate specific substrates through other types of catalysis, including halogen‐bonding catalysis,^[14]^ Brønsted base catalysis^[15]^ and photoredox catalysis,[^16^, ^17^, ^18^] among others. As concerning enamine catalysis, an amine can easily condensate with an α‐enolizable carbonyl compound, resulting in the formation of the corresponding imine. Subsequently, through tautomeric equilibrium, the imine is converted into its enamine derivative. This results in the energy raising of the highest occupied molecular orbital (HOMO), ultimately increasing the nucleophilicity of the reactive intermediate (enamine) if compared to the enol form of the starting material.^[19]^ Similarly, amine catalyst can condensate with an α,β‐unsaturated carbonyl compound, in the presence of an acidic co‐catalyst, resulting in the formation of an iminium ion. In this intermediate, the lowest unoccupied molecular orbital (LUMO) is energetically lowered compared to that of the starting carbonyl compound, hence facilitating the nucleophilic attack to the β‐carbon.^[20]^ Besides, another key aspect of organocatalysis is the ease of developing novel asymmetric transformations.^[13]^ In fact, in recent years, chiral and enantiopure aminocatalysts have been effectively employed to drive the formation of a wide variety of enantioenriched organic products.^[21]^ It is worth noting that molecular aminocatalysts are a class of bio‐inspired catalytic systems, since a number of biological active enzymes bear amine‐rich functional groups in their active sites.^[13]^

This Concept aims at illustrating how the detailed understanding of the nature, amount, and reactivity of the amino groups on CD surface allows for the development and design of novel green nano‐aminocatalytic platforms, connecting between the molecular level and the nanoscale domain.

## 2 Synthesis, Purification and Characterization of Nitrogen‐Doped CDs

**Figure 2:**
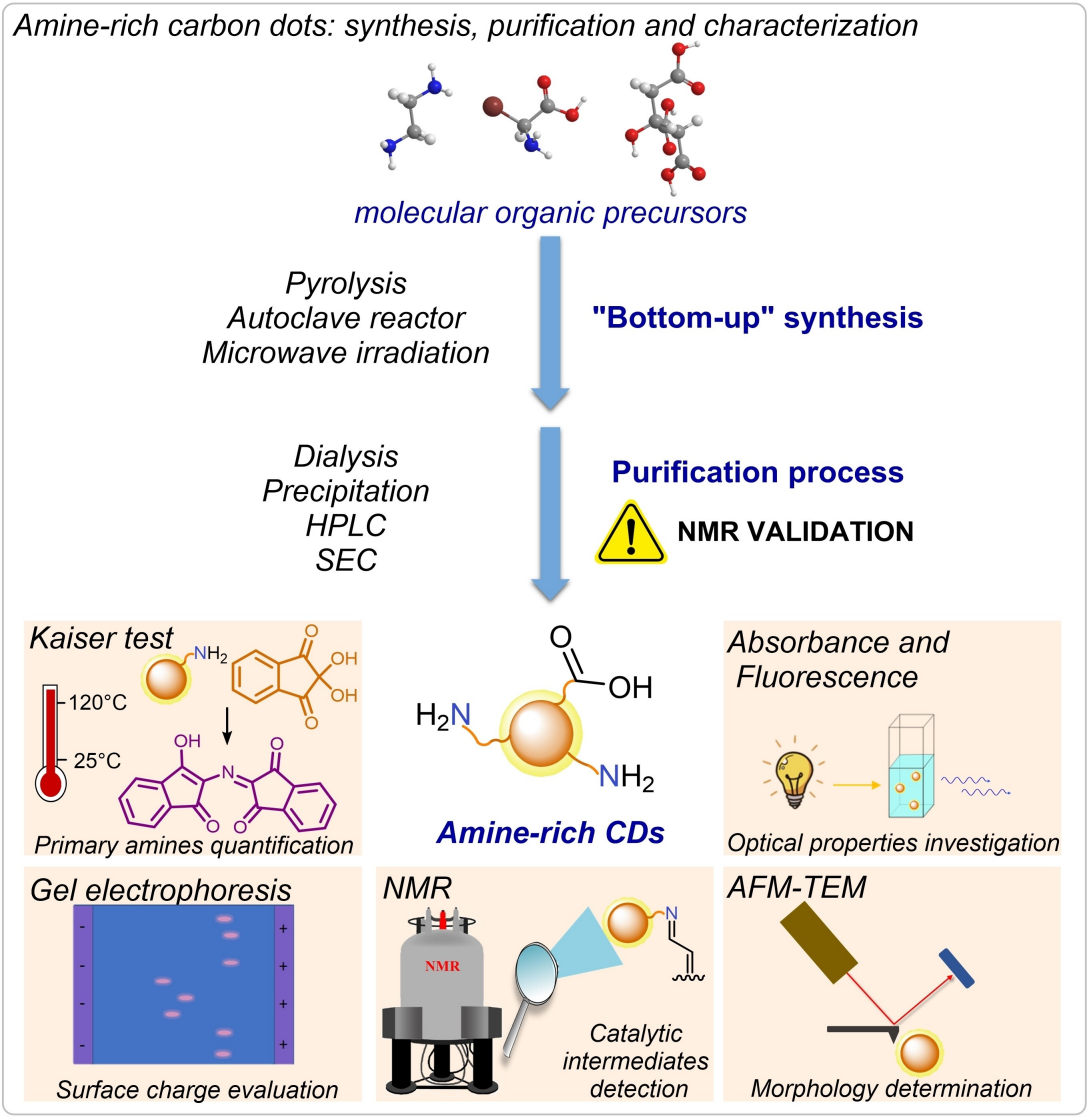
The “bottom‐up” treatment of molecular precursors followed by the purification process to yield CDs; recurrent characterization techniques used to analyze these amine‐rich nanoparticles.

The main synthetic strategies for the preparation of CDs may be classified as “top‐down” or “bottom‐up”. In particular, “bottom‐up” approaches enable the production of a wide variety of CDs by solvothermal treatments or pyrolysis of simple and readily available molecular precursors.^[22]^ Operationally, these syntheses are performed in autoclave or microwave (MW) vessel, where the careful control of the temperature furnishes different features of the final material (Figure 2). In particular, low‐temperature treatments (T<300 °C) result, typically, in the formation of amorphous CDs, whereas heating at higher temperature (T>300 °C) favors their graphitization.^[23]^ Usual substrates used for the CD preparation are citric acid (CA), amino acids, and small aromatic molecules, such as anilines and phenol derivatives, which are abundant and inexpensive compounds able to undergo condensation, dehydration and decarboxylation reactions under thermal treatments.^[24]^ Moreover, the introduction of a second component in the CD preparation (e. g., amines, thiols, phosphonates, borates, etc.), which can act as passivating agent, has proven to be an effective tool in the synthesis of hetero‐atom doped nanoparticles, with numerous reactive functionalities on their surface and improved optical properties.^[3]^ Furthermore, an appealing trait of the “bottom‐up” synthesis is that part of the properties of the reagents could be retained in the nanoparticles structure.^[25]^ As examples, it was possible to envision the production of chiral CDs starting from *ad hoc* enantiopure chiral organic compounds,[^26^, ^27^] or electrochemically‐rich CDs starting from quinones.^[28]^

Despite the straightforward preparation and the versatility of the synthetic protocols, the purification of CDs can be nontrivial.^[29]^

Indeed, the high temperature treatment employed in the synthesis enables multiple reaction pathways resulting in the formation of CDs along with small molecular weight side‐products. The effective removal of these species is extremely important: in fact, the presence of residual organic molecules can affect the properties of the final materials.^[30]^ Depending on the solubility of the nanoparticles different purification techniques may be employed, such as dialysis, precipitation, high‐performance liquid chromatography (HPLC), size‐exclusion chromatography (SEC) among others. In this context, our group has recently demonstrated that a valuable technique to confirm the effective purification of CDs is the use of nuclear magnetic resonance (NMR) spectroscopy (Figure 2).^[31]^

The in‐depth characterization of both the carbon cores and the surface groups of the purified CDs is also a matter of paramount importance. The dimensions of the nanoparticles can be derived from atomic force microscopy (AFM) or transmission electron microscopy (TEM). Moreover, employing TEM, Raman spectroscopy and thermogravimetric analysis (TGA) it is possible to get insights on the chemical structure of the CDs, thus distinguishing between amorphous and graphitic nanoparticles.^[32]^ Afterwards, absorption and emission spectroscopies can be employed to investigate the optical properties of the CDs. Specifically, most CDs display a broad absorption in the visible region of the spectrum together with an excitation‐dependent emission profile and a large Stokes shift.^[33]^

It is evident that a detailed surface characterization of the materials is crucial to enable their use as nano‐organocatalysts.^[34]^ X‐ray photoelectron spectroscopy (XPS) and infrared spectroscopy (IR) are often employed to uncover the elemental composition and the nature of the surface groups. Additionally, agarose gel electrophoresis can be performed to gain insights on the nanoparticle surface charge at a specific pH‐value. Afterward, it is possible to estimate the total number of acid/base sites using the Gran Plot analysis, which consists of a linearization of a simple pH back titrations.^[35]^

The exploitation of amine‐rich CDs as nano‐organocatalysts is directly linked to the mapping of the surface amino groups. Generally, the first analysis carried out is the Kaiser test (KT).^[29]^ KT is a colorimetric test used to detect and quantify the presence of primary aliphatic amines. This method relies on the condensation of amines with ninhydrin at 120 °C, forming an imine intermediate that proceeds further with the formation of an organic dye, namely Ruhemann's purple (Figure 2). Moreover, a modified KT procedure can be employed to estimate the accessibility of these functional groups. Indeed, performing KT at ambient temperature, instead of 120 °C, provides insight into the reactivity and availability of the surface amino groups.^[34]^ In addition, secondary and aromatic amines, which are not detectable using KT analysis, remain of prominent interest as aminocatalytic sites. In order to obtain additional information on this aspect, it is possible to study the surface groups of CDs by NMR upon formation of covalent adducts.^[34]^ In fact, the in‐situ condensation of model carbonyl compounds with the surface amines of CDs generates the corresponding imine, iminium‐ion or enamine species. This enables the direct detection of the different aminocatalytic intermediates as a fingerprint of the surface functional groups.

## 3 Use of Amine‐Rich CDs in Organocatalysis

**Figure 3:**
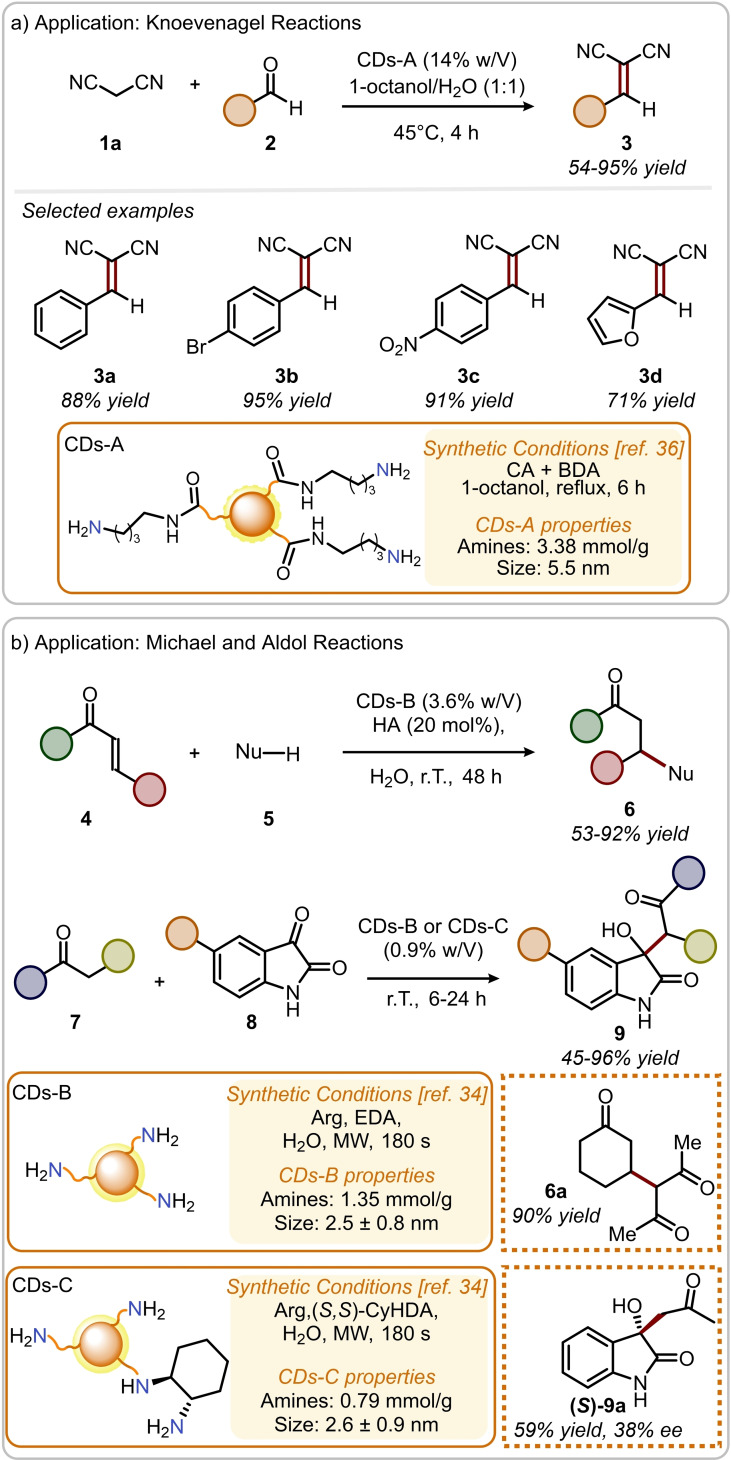
a) Application of CDs‐A in the Knoevenagel reactions; b) application of CDs‐B and CDs‐C in aldol‐type reactions and Michael additions.

CDs bearing amino groups on their surface are capable of driving aminocatalytic organic reactions on suitable carbonyl substrates. In 2018 Wang and co‐workers reported the use of amine‐terminated CDs‐A (Figure 3a) to promote the Knoevenagel condensation of malononitrile **1 a** and benzaldehydes **2** in a water/1‐octanol biphasic system at 45 °C.^[36]^ These carbon‐based materials were synthesized by pyrolysis of CA and a suitable diamine in 1‐octanol, at 180 °C, for 6 h, under inert atmosphere. The authors demonstrated that the choice of the diamine precursor enabled the synthesis of CDs featuring aliphatic primary, secondary, tertiary amines on their surface. However, primary amine‐terminated CDs‐A resulted in improved catalytic activity (compared to secondary and tertiary amines) for the desired Knoevenagel condensation and were thus selected for the evaluation of the reaction scope, providing the arylidene malononitriles **3** in generally very good yields (Figure 3a, 54–95 %). Interestingly, CDs‐A could be transferred to the aqueous phase upon bubbling CO_2_ into the reaction mixture, owing to the formation of carbamates or ammonium bicarbonates on their surfaces. After filtration of the product from the organic phase, the solubility of CDs‐A was switched by bubbling a stream of N_2_ to remove CO_2_ and restore the functional groups on their surfaces. The reversible phase‐transfer of CDs‐A by means of CO_2_ is an elegant approach towards sustainable nano‐organocatalysis and allows the recycling of the catalyst in an efficient way.

The study and characterization of the functional groups on the surface of CDs are of great importance to envision their future applications. For example, our group carried out the in‐depth topological studies of the amino groups on the surface of CDs‐B (Figure 3b), prepared by hydrothermal treatment from L‐Arginine (Arg) and ethylenediamine (EDA). These carbon‐based nanoparticles were applied as water‐soluble aminocatalysts for a variety of organic reactions.^[34]^ The presence of superficial primary and secondary amino groups was leveraged applying the two most classical activation modes in aminocatalysis: the HOMO‐raising and the LUMO‐lowering approach. The covalent formation of iminium ion intermediates in water media was exploited to promote Michael addition reactions of various nucleophiles to cynnamaldehydes or α,β‐unsaturated cyclic ketones, obtaining good to excellent yields (Figure 3b, 53–92 %). Importantly, the formation of reactive iminium ion intermediates on the surface of CDs‐B was observed by ^19^F NMR using 4‐fluorocinnamaldehyde as molecular fluorinated probe. In fact, NMR is emerging as promising technique to study the reaction mechanisms of these nano‐organocatalytic transformations, as it is capable of detecting and quantifying the formation of reactive intermediates on CDs. Furthermore, CDs‐B were also used to form electron rich enamine intermediates and catalyze the aldol‐type addition of various ketones to isatines, providing the corresponding oxindole derivatives in most cases in excellent yields (Figure 3b, 45–96 %). Finally, the synthesis of CDs‐C (Figure 3b) from Arg and (*S*,*S*)‐1,2‐cyclohexanediamine ((*S*,*S)‐*CyHDA) gave rise to nanoparticles with chiral amines on their surface.^[6]^ Importantly, the choice of the appropriate precursors (for instance, chiral cyclic diamines), that retain their chirality at high temperatures, is essential in designing the synthesis of new chiral carbon dots. CDs‐C were used for the asymmetric catalytic aldol addition of acetone to isatine, producing the desired product in 59 % yield and 38 % ee when CH_2_Cl_2_ was used as solvent. This is probably one of the first examples of asymmetric synthesis at the nanoscale, where the chirality of the carbon‐based nanoparticle was transferred to the desired product.

**Figure 4:**
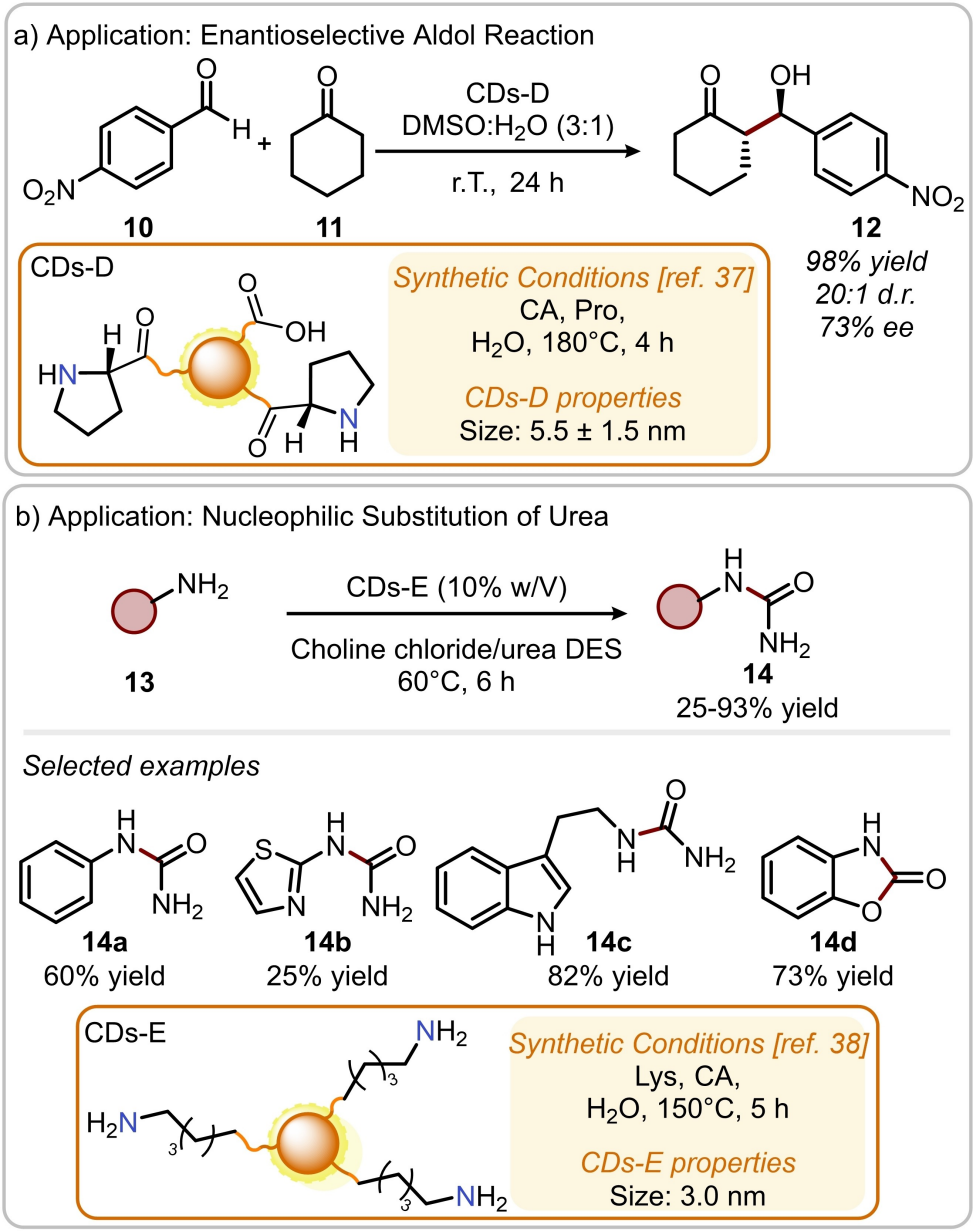
a) Application of CDs‐D in the aldol addition of cyclohexanone to 4‐nitrobenzaldehyde; b) application of CDs‐E in the nucleophilic substitution of urea.

Recently, it was reported the synthesis of CDs‐D (Figure 4a) from a combination of CA and L‐Proline (Pro) by hydrothermal treatment at 180 °C for 4 h.^[37]^ These carbon nanoparticles were used as catalysts to promote the direct catalytic asymmetric aldol reaction between cyclohexanone **11** and 4‐nitrobenzaldehyde **10**. The aldol adduct **12** was obtained in excellent yield (98 %), d.r. (20 : 1) and good ee (73 %); these results are comparable to those obtained with pure L‐Proline (96 % yield, 20 : 1 d.r. and 93 % ee). Despite the thorough characterization of CDs‐D and the control experiments carried out to exclude the presence of uncarbonized L‐Proline, the authors did not report the NMR characterization of CDs‐D that would exclude completely the presence of molecular species adsorbed to the carbon‐based material.^[31]^ Furthermore, for the catalytic asymmetric aldol reaction, the catalyst loading was not reported.

Another example on the use of nitrogen‐doped CDs to catalyze organic reactions was reported recently by Kalhor and co‐workers.^[38]^ This study focuses on the synthesis, characterization and application in catalytic reactions of nitrogen‐doped CDs‐E (Figure 4b). The nanoparticles were prepared from CA and L–Lysine (Lys) that was specifically chosen because of its long carbon chain terminated with a primary amine. This feature was expected to improve the catalytic activity of CDs‐E as the long chain renders the primary amine more available to activate the given substrate. More specifically, CDs‐E were employed to catalyze the nucleophilic substitution of urea with (hetero)aromatic and aliphatic amines **13** at 60 °C in choline chloride/urea as deep eutectic solvent (DES) system, which was found to be essential for reactivity. The scope of the reaction showed a broad tolerance with regards of anilines, affording the corresponding monosubstituted ureas **14** in yields ranging from 45 % to 75 %. When primary aliphatic amines were used in the reaction, the yields improved sensibly (82–93 %). Interestingly, it was possible to use the same catalytic system to achieve the synthesis of 2‐benzoxazolinone **14 d** (73 % yield), starting from 2‐aminophenol, in a catalytic cascade process.

**Figure 5:**
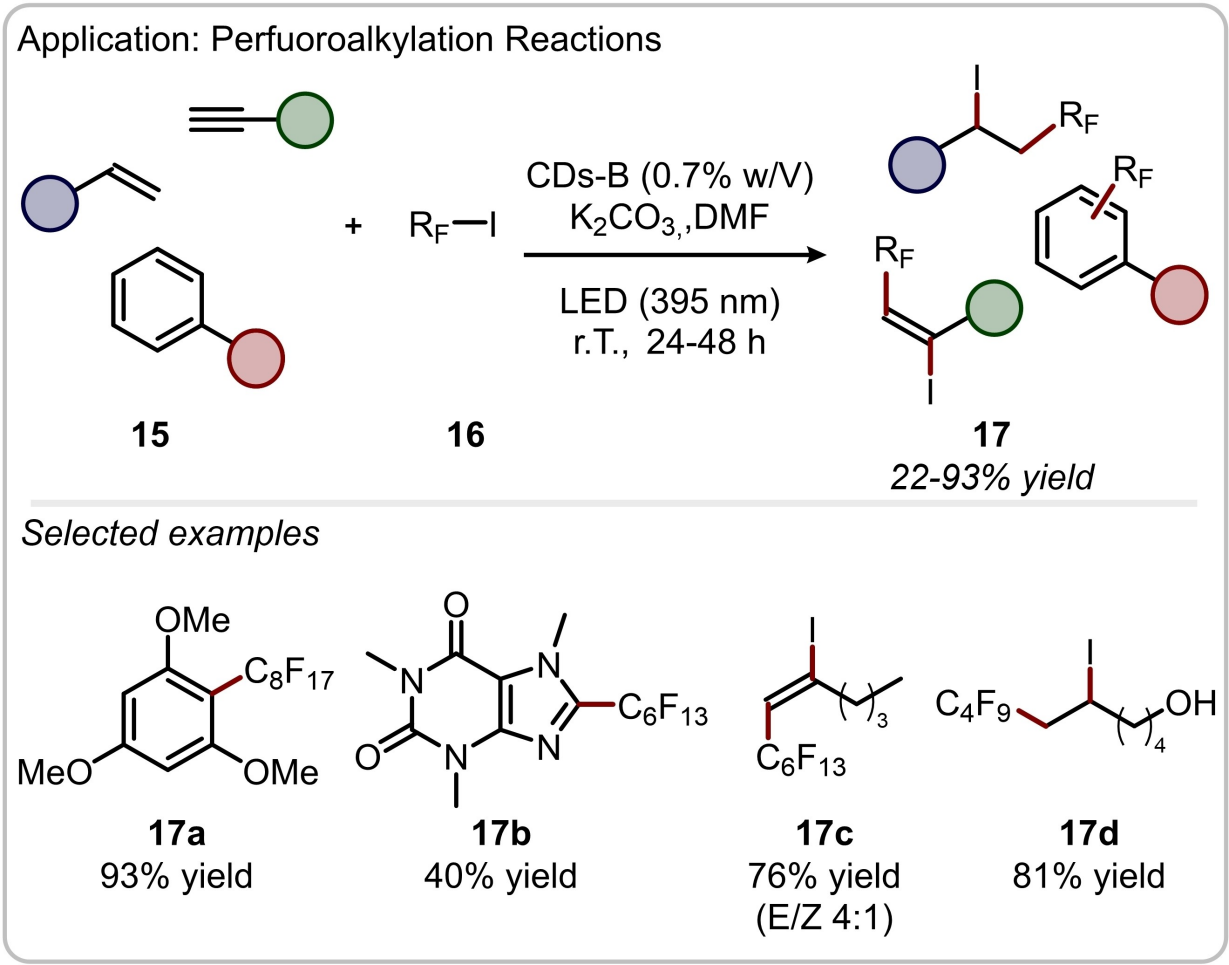
Application of CDs‐B in the fluoroalkylation of unsaturated organic compounds.

The application in catalysis of *N*‐doped CDs is not only limited to those reactions that rely on the canonical activation modes of aminocatalysis. Indeed, the optical properties of these materials lay the foundations for their application as green photocatalytic platforms for light‐driven transformations in organic chemistry. For example, in 2019 we reported that CDs‐B (Figure 3b) can efficiently promote the photochemical perfluoroalkylation of unsaturated organic compounds.^[39]^ More in detail, upon visible light absorption (395 nm), CDs‐B can reach their excited state and undergo a single‐electron transfer (SET) event to photo‐reduce perfluoroalkyl iodides **16** and trigger the generation of open shell intermediates. This reactive species can be then trapped by a variety of unsaturated organic substrates **15** to deliver perfluorinated products **17** in up to 90 % yield (Figure 5). It is worth noting that the primary amino groups on the surface of CDs‐B, which derive from Arg and EDA as synthetic precursors, are able to interact with the perfluoroalkyl iodide *via* halogen‐bond association, bringing in close proximity the radical source and the CDs to facilitate the photochemical initiation process.

## 4 Conclusions and Future Perspectives

In recent years, amine‐rich CDs have been exploited as effective nano‐catalytic platforms for a wide range of organic reactions. Specifically, this Concept focuses, through the analysis of the most explicative examples, on the current progress toward the preparation and application of amine‐bearing CDs as catalysts to promote transformations of synthetic importance. Despite the promising results achieved to date, a large number of unexplored opportunities and challenges remain unaddressed. We foresee that the advancement within this field will require the development of novel and increasingly active CDs‐based catalysts. In this direction, a deep understanding of the formation processes of CDs and, in particular, the origin of their surface amino functionalities has to be clarified. Besides, enantioselective nano‐aminocatalysis in water or even in biological media should be achieved by using chiral and optically active CDs. This could lead to the development of numerous green nano‐aminocatalytic methodologies for the synthesis of valuable enantio‐enriched organic molecules under mild operative conditions. In principle, chiral CDs produced from amino acids may mimic the chemical behavior of enzymes. This general concept is known as nanozymes and might result in *in vivo* clinical applications, including diagnostics, therapeutics and drug synthesis^[9]^ Moreover, further stereocontrolled reactions should be achieved by merging the organocatalytic and photocatalytic aptitudes of chiral amine‐rich CDs. In fact, CDs can be used to produce open‐shell species from suitable radical precursors under visible‐light irradiation. Afterwards, these reactive radical intermediates might be trapped by the chiral aminocatalytic intermediates, namely iminium ions or enamines, formed on the surface of the nanoparticles, ideally in an enantioselective fashion. Lastly, it is important to mention that the recyclability of the CD‐based aminocatalysts may not be trivial. Thus, effective strategies (e. g., centrifugation, precipitation, extraction with a suitable solvent, among other) for the reuse of these amine‐rich nanoparticles have to be developed. To conclude, we believe that, in the future, more thorough investigations, both on the chemical nature and on the reactivity of the surface amino groups of such CDs will help solve the present‐day challenges in organic synthesis.

## Conflict of interest

The authors declare no conflict of interest.

## Biographical Information

*Vasco Corti received his Ph.D. in chemistry in 2019 at the ‘Toso Montanari’ department of the University of Bologna (Italy) under the supervision of Prof. Luca Bernardi and Prof. Mariafrancesca Fochi. He spent two years as a post‐doctoral researcher at Aarhus University (Denmark) under the supervision of Prof. Karl Anker Jørgensen working on the development of new aminocatalytic enantioselective higher‐order cycloadditions as well as the development of novel aminocatalytic atroposelective reactions. He is currently conducting postdoctoral studies in the group of Prof. M. Prato and his research focuses on the development of new photochemical processes and the study of new chiral carbon dots and their application in catalysis*.

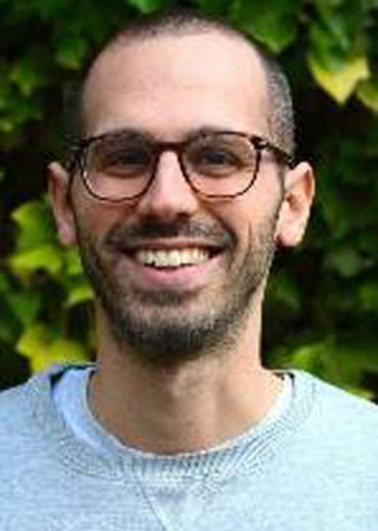

## Biographical Information

*Beatrice Bartolomei obtained her Master's degree in Chemistry from the University of Perugia (Italy) in 2020. During the same year, she started her doctoral studies in the group of Prof. Maurizio Prato at University of Trieste (Italy), focusing her research on the study of the physicochemical properties of carbon‐based nanomaterials*.

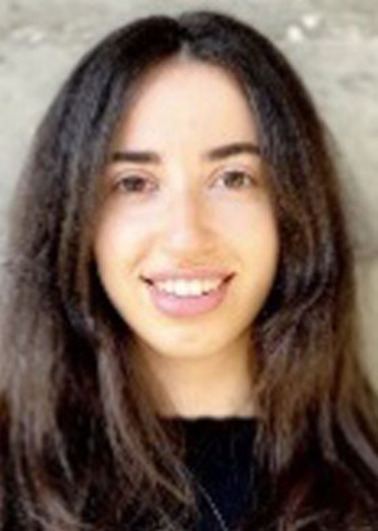

## Biographical Information

*Martina Mamone obtained her Master's degree in Industrial Chemistry from the University of Bologna (Italy) in 2021. During the same year, she started her doctoral studies in the group of Prof. Maurizio Prato at University of Trieste (Italy), working on the use of carbon dots as nano‐organocatalysts in synthetic chemistry*.

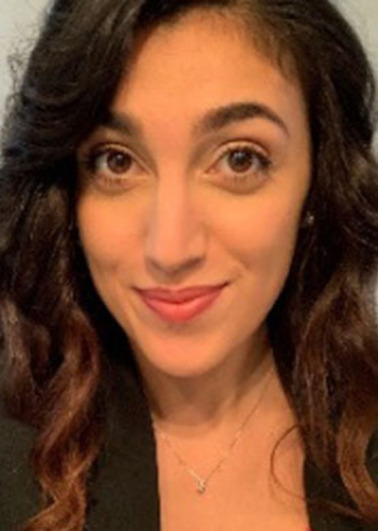

## Biographical Information

*Giuseppe Gentile received his Master's degree in chemistry in 2019 from the University of Calabria (Italy). After graduation he joined the group of Prof. Luisa De Cola at the Institut de Science et d′Ingénierie Supramoléculaires (ISIS) in Strasbourg (France) for a short‐term research stay. In fall of 2020, he joined the group of Prof. Maurizio Prato at University of Trieste for PhD studies. He currently works on the synthesis and applications of carbon‐based nanomaterials for organo‐ and photocatalysis*.

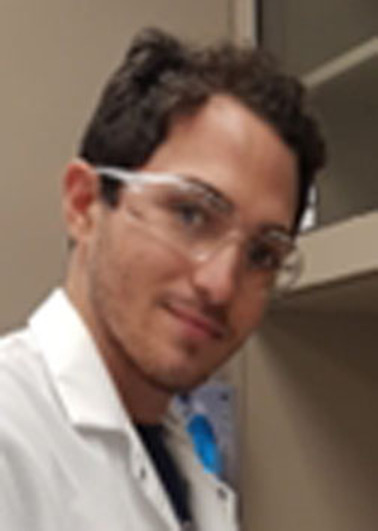

## Biographical Information

*Maurizio Prato is Professor of Organic Chemistry at the University of Trieste and Ikerbasque Research Professor at CIC biomaGUNE, Spain. He was the recipient of two ERC Advanced Research Grant, European Research Council, in 2008 and 2020 and became a Member of the National Academy of Sciences (Accademia Nazionale dei Lincei) in 2010. His research focuses on the synthesis of innovative carbon‐based functional materials, for applications in materials science, nanomedicine, and catalysis*.

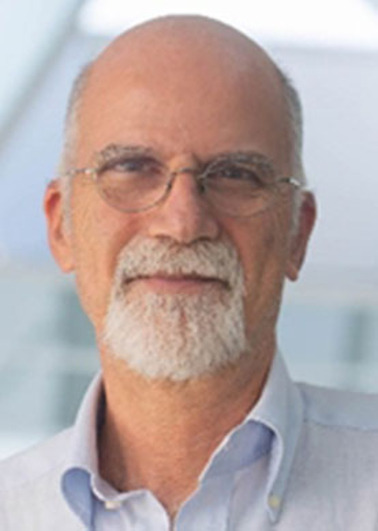

## Biographical Information

*Giacomo Filippini obtained his Master's degree in Industrial Chemistry from the University of Bologna (Italy). In 2013 he joined the group of Prof. Paolo Melchiorre at ICIQ in Tarragona (Spain), where he undertook his doctoral studies. In 2017, he started a postdoctoral appointment in the group of Prof. Maurizio Prato at University of Trieste (Italy), where he is currently Assistant Professor, investigating the use of carbon‐based nanomaterials to design novel organic transformations*.